# A Randomized Controlled Trial of a Psycho-Education Intervention by Midwives in Reducing Childbirth Fear in Pregnant Women

**DOI:** 10.1111/birt.12136

**Published:** 2014-10-09

**Authors:** Jocelyn Toohill, Jennifer Fenwick, Jenny Gamble, Debra K Creedy, Anne Buist, Erika Turkstra, Elsa-Lena Ryding

**Affiliations:** Griffith Health Institute, School of Nursing & Midwifery, Griffith UniversityBrisbane, Qld, Australia; Gold Coast University HospitalParkwood, Qld, Australia; University of MelbourneMelbourne, Vic, Australia; Department of Women's and Children's Health, Division of Obstetrics and Gynecology, Karolinska InstitutetStockholm, Sweden

**Keywords:** childbirth fear, midwife counseling, psycho-education, childbirth self-efficacy, depression, decisional conflict, RCT

## Abstract

**Background:**

Childbirth fear is associated with increased obstetric interventions and poor emotional and psychological health for women. The purpose of this study is to test an antenatal psycho-education intervention by midwives in reducing women's childbirth fear.

**Methods:**

Women (*n* = 1,410) attending three hospitals in South East Queensland, Australia, were recruited into the BELIEF trial. Participants reporting high fear were randomly allocated to intervention (*n* = 170) or control (*n* = 169) groups. All women received a decision-aid booklet on childbirth choices. The telephone counseling intervention was offered at 24 and 34 weeks of pregnancy. The control group received usual care offered by public maternity services. Primary outcome was reduction in childbirth fear (WDEQ-A) from second trimester to 36 weeks’ gestation. Secondary outcomes were improved childbirth self-efficacy, and reduced decisional conflict and depressive symptoms. Demographic, obstetric & psychometric measures were administered at recruitment, and 36 weeks of pregnancy.

**Results:**

There were significant differences between groups on postintervention scores for fear of birth (*p* < 0.001) and childbirth self-efficacy (*p* = 0.002). Decisional conflict and depressive symptoms reduced but were not significant.

**Conclusion:**

Psycho-education by trained midwives was effective in reducing high childbirth fear levels and increasing childbirth confidence in pregnant women. Improving antenatal emotional well-being may have wider positive social and maternity care implications for optimal childbirth experiences.

High rates of childbirth fear have been reported in Scandinavian countries 1–5, Australia 6–8, and the United Kingdom 9. Childbirth fear has been linked to adverse maternal outcomes including poor postpartum mental health and high rates of cesarean 10–15. Outside Sweden, no reported systematic approach was seen to identify and care for fearful women 16,17. Without appropriate intervention, women reporting high fear may perceive surgery as their only birth option 18. Although intervention studies have focused on improving vaginal birth rates, no trials have reported on the effectiveness of an antenatal intervention to reduce fear levels before birth 19,20. This paper reports on the effects of an intervention to reduce fear in pregnant women and assist their preparation for a positive birth.

## Aim

To test an antenatal psycho-education counseling intervention by midwives in reducing women's childbirth fear.

## Hypotheses

Relative to women in the control group, women receiving the psycho-education intervention will report lower levels of childbirth fear at 36 weeks; improved birth confidence, less decisional conflict; and less depressive symptoms.

## Method

Participants were drawn from a multisite randomized controlled trial. The protocol for the study (known as BELIEF: Birth Emotions: Looking to Improve Expectant Fear) has been published 21. A two arm nonblinded parallel trial design was used. Participants were stratified by hospital site and parity and allocated to study groups using a web-based randomization service to generate blocks for groups of ten. A research assistant accessed the service after receiving a participant's written consent and completed baseline measures. A midwife providing the intervention was notified of the woman's contact details. Ethical approval was obtained from the university and participating hospitals.

## Participants and Setting

Women in their second trimester attending antenatal clinics of three hospitals in South East Queensland, Australia, able to communicate sufficiently in English, and aged 16 years or older were recruited by research assistants. Participants were screened for high childbirth fear using the Wijma Delivery Expectancy/Experience Questionnaire Version A (W-DEQ A) 22. Women requiring an interpreter, younger than 16 years, or more than 24 weeks pregnant, and anticipating or experiencing a perinatal death (e.g., congenital abnormality incompatible) or stillbirth were excluded. Participants (*n* = 1,410) were recruited from May 2012 to June 2013. Three hundred and thirty-nine women (*n* = 339) reporting high fear (defined as a score of ≥ 66 on the W-DEQ A) were allocated to the intervention (*n* = 170) or control (*n* = 169) groups. Two women were incorrectly randomized (W-DEQ A scores < 66) and removed from the analysis. This paper reports on primary and secondary antenatal outcomes for women who returned data at 36 weeks’ gestation. Baseline characteristics of participants were similar to the national Australian birthing population and have been reported 8.

## Sample Size

The sample size was determined on a reduction in level of fear between the intervention group and the control group. The statistical package R-Project version 2.14.2 23 was used to calculate a meaningful reduction in level of childbirth fear based on the study of Fenwick, Gamble, Nathan, Bayes, and Hauck 6 who found one in four women experienced childbirth fear. The calculation also included a standard error of measure to determine how an individual's own score would change over time. A standard deviation of 20 and reliability of 0.87 based on the W-DEQ A 2,22 provided a standard error of measurement to be 7.211. For an 80 percent chance to detect a 10-point difference in WDEQ scores from baseline to around 36 weeks’ gestation in the intervention compared with the control group, 140 women plus 30 percent allowance for attrition were needed. The estimate was based on a two-tailed *α* = 0.05.

## Measures

**Figure 1:**
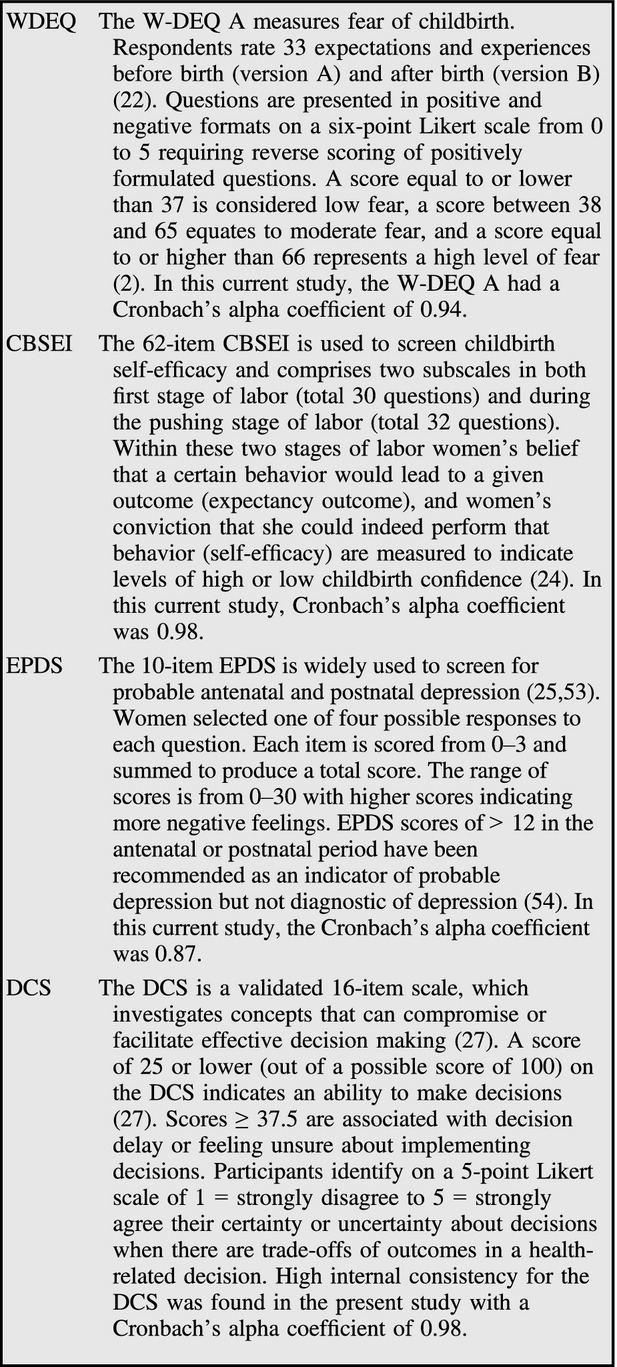
Validated measures used in the study.

Demographic, obstetric details, and birth preference were collected. The primary outcome measure was a reduction in childbirth fear as measured by the W-DEQ A. Secondary outcomes measures included the Childbirth Self-Efficacy Inventory (CBSEI) 24; Edinburgh Postnatal Depression Scale (EPDS) 25, which has been validated for use in pregnancy 26; and the Decisional Conflict Scale (DCS) 27 (Fig.1). Data were collected at recruitment and 36 weeks of pregnancy.

## Intervention

BELIEF is a telephone psycho-education counseling intervention offered by midwives. The intervention aims to review women's current expectations and feelings around fear of childbirth, support the expression of feelings, and provide a framework for women to identify and work through distressing elements of childbirth. The intervention was adapted from a midwifery counseling framework for distressed postpartum women developed by Gamble and Creedy 28. The BELIEF intervention aimed to help women develop individual situational supports for the present and near future, affirming that negative things can be managed with a simple plan developed to achieve this. Women in the control group received usual care offered by publicly funded maternity services in Australia. All participants received a copy of a decision-aid booklet titled *Having a baby in Queensland* 29.

## Statistical Methods

Data were analyzed using the Statistical Package for the Social Sciences (SPSS) version 21.0 for Windows 30. An alpha level of 0.05 was used for all statistical tests. A one-way between-groups analysis of covariance compared the effectiveness of the intervention in reducing the primary outcome of childbirth fear (W-DEQ A). The independent variable was group allocation, and dependent variable was participants’ fear score. Women's baseline fear score at recruitment was used as the covariate.

Secondary outcomes were conducted using independent samples *t*-test. Change scores for fear (W-DEQ A), decisional conflict (DCS), and depression (EPDS) were calculated (Time 1−Time 2) for both groups comparing posttreatment scores for the intervention and control groups with the baseline score as the covariate. Calculation of change scores for childbirth confidence (CBSEI) were Time 2−Time 1 because of inverse scoring compared with the other measures. Effect size statistics (Cohen's d) were determined by subtracting the mean change score for the two groups and dividing by the pooled baseline SD 31. Furthermore, the chi square statistic compared percentages of cases in the intervention and control groups who improved or showed no improvement/deteriorated for secondary outcomes to illustrate individual response to the treatment rather than mean scores for each group. Bonferroni adjustment was used to determine significance levels for group comparisons 32.

## Results

No significant differences were reported between women returning all data (*n* = 198) and women lost to follow-up (*n* = 141) for age, country of birth, parity, previous mode of birth, marital status, DCS, EPDS, CBSEI, and W-DEQ A scores. However, statistically significant differences were found for education and household income (Table1). Women lost to follow-up were less likely to be educated beyond Year 12, and less likely to have a household income of more than $AUD 52,000 per annum in comparison to women who completed the study.

**Table 1:** Comparison of Randomized Participants Who Returned Data Versus Those Who Did Not Return Data

Characteristics	Participants returning data at 36 weeks *n *= 198	Participants not returning data at 36 weeks *n *= 141	*p*
	No. (%)	No. (%)	
Age (years) Mean [SD, range]	29.1 [5.44, 17–51]	27.9 [5.89, 17–40]	0.06
No partner	11 (5.6)	15 (10.6)	0.13
Education Year 12 or less	84 (42.4)	82 (58.2)	0.006
Income			
$0–$51,999	53 (26.8)	55 (39)	0.04
$52,000–$77,999	55 (27.8)	34 (24.1)	
> $78,000	85 (42.9)	46 (32.6)	
Missing	5 (2.5)	6 (4.3)	
Foreign born	56 (28.3)	35 (24.8)	0.56
Aboriginal	4 (2)	4 (2.8)	0.90
Nulliparous	116 (58.6)	75 (53.2)	0.38
Cesarean last birth			
No labor/Cesarean	9 31	10 (43.5)	0.52
Labor/Cesarean	20 (69)	13 (56.5)	
Preferred mode of birth			
Cesarean	31 (16.1)	23 (16.4)	1.00
Missing	4 (2)	1 (< 1)	
W-DEQ Mean [SD, range]	78.3 [11.8, 66–127]	77.8 [11.5, 63–128]	0.69
Missing	3 (1.5)	4 (2.8)	
CBSEI Mean [SD, range]	377 [113.4, 62–620]	372 [126.5, 62–620]	0.71
Missing	6 (3)	9 (6.4)	
DCS Mean [SD, range]	40.23 [22.11, 0–100]	40.32 [23.29, 0–100]	0.89
Missing	2 (1)	3 (2)	
EPDS Mean [SD, range]	8 [5.35, 0–24]	7.7 [5.22, 0–23]	0.65

W-DEQ = Wijma Delivery Expectancy Scale; CBSEI = Childbirth Self-Efficacy Inventory; DCS = Decisional Conflict Scale; EPDS = Edinburgh Postnatal Depression Scale.

**Figure 2:**
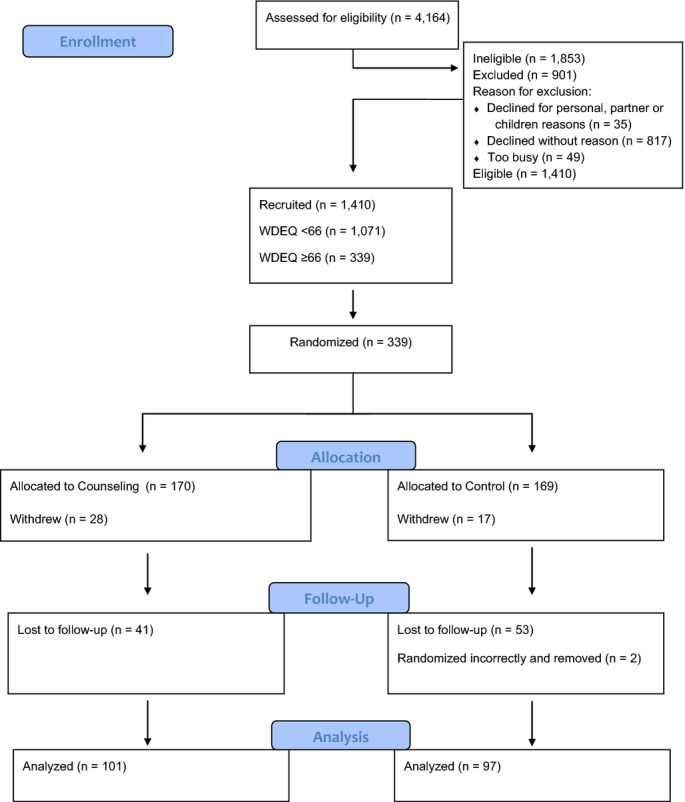
The Consolidated Standards of Reporting Trials (CONSORT) flow diagram.

One hundred and ninety-eight (*n* = 198, 58%) eligible women completed the follow-up questionnaire at 36 weeks. Baseline comparison of women randomized to intervention and control arms of the study are presented in Table2. One hundred and thirty-nine (*n* = 139) women did not complete the second questionnaire; of these, 45 withdrew (2 because of late pregnancy loss), and 94 were lost to follow-up (including four who gave birth prematurely). The Consolidated Standards of Reporting Trials (CONSORT) participant flow diagram is presented in Fig.2.

**Table 2:** Baseline Participant Characteristics for Treatment and Control Groups

Characteristic	Randomized to treatment group *N *= 101	Randomized to control group *N *= 97
	No. (%)	No. (%)
Age (years) Mean [SD, range]	29 [5.9, 17–51]	29.2 [4.98, 18–42]
Education Year 12 or less	49 (48.5)	35 (36.1)
Income		
0–$51,999 per annum	24 (23.8)	29 (29.9)
> $52,000–$77,999	35 (34.7)	20 (20.6)
> $78,000 per annum	41 (40.6)	44 (45.4)
Missing	1 (1)	4 (4.1)
Nulliparous	58 (57.4)	58 (59.8)
No partner	9 (8.9)	2 (2.1)
Foreign born	25 (24.8)	31 (32)
Gestation at recruitment Mean [SD, range]	18.2 [3.17, 11–25]	17.9 [2.8, 13–24]
Preferred birth mode		
Cesarean	15 (14.9)	16 (16.5)
Uncertain	2 (2)	3 (3.1)
Cesarean last birth		
No labor cesarean	5 (5)	4 (4.1)
Labor/Cesarean	10 (9.9)	10 (10.3)
History depression/anxiety	52 (51.5)	42 (43.3)
History tobacco smoking	52 (51.5)	58 (59.8)
W-DEQ Median {IQR}	77 {72,86}	73 {69, 78.5}
W-DEQ Mean [SD, range]	80.9 [13.1, 66–127]	75.7 [9.7, 66–115]
CBSEI Median {IQR}	372 {301, 465.5}	371.5 {302, 464}
CBSEI Mean [SD, range]	368.5 [122.5, 62–620]	385.9 [102.9, 62–591]
Missing	3 (3)	3 (3)
EPDS Median {IQR}	9 {4, 13}	7 {3.5, 10}
EPDS Mean [SD, range]	8.72 [5.8, 0–22]	7.33 [4.7, 0–24]
DCS Median {IQR}	43.7 {25, 56.2}	37.5 {26.6, 50}
DCS Mean [SD, range]	41.3 [24.3, 0–100]	39.1 [19.5, 0–94]
Missing	–	2 (2)

W-DEQ = Wijma Delivery Expectancy Scale; CBSEI = Childbirth Self-Efficacy Inventory; DCS = Decisional Conflict Scale; EPDS = Edinburgh Postnatal Depression Scale; IQR = Interquartile range: 25th percentile, 75th percentile.

Of the 170 women allocated to the intervention group, 101 (59.4%) returned data. Of these, three women (2.9%) did not receive the intervention, six women (5.9%) completed one session, and 92 (91%) received both sessions of the intervention as planned. The mean duration of the first psycho-education session was 58 minutes (range = 22–125 minutes) and 45 minutes for the second session (range = 10–104 minutes).

### Internal Reliability of Scales

Reliability of measures was assessed using baseline scores. Cronbach's alpha coefficient for each scale indicated good internal consistency (as shown in Table3).

**Table 3:** Internal Reliability of Scales

Scale	Cronbach's alpha	Mean [SD, Range]
Wijma Delivery Expectancy/Experience Questionnaire (W-DEQ-A)	0.93	49.5 [21.9, 0–128]
ChildBirth Self-Efficacy Inventory (CBSEI)	0.98	443 [112.2, 62–620]
Edinburgh Postnatal Depression Scale (EPDS)	0.86	5.1 [4.6, 0–24]
Decisional Conflict Scale (DCS)	0.97	29.7 [23, 0–100]

### Effect of the Intervention on Childbirth Fear

After adjusting for preintervention scores, a significant difference resulted between groups on postintervention W-DEQ A scores for fear of birth, (*F*(1, 191) = 11.6, *p* = 0.001, partial eta squared = 0.06) with medium effect 33. Women receiving the intervention reported reduced childbirth fear at 36 weeks compared to women in the control group.

### Outcome Measurement Change Scores

Change scores for the primary outcome of fear (W-DEQ A) and the secondary outcomes of childbirth self-efficacy (CBSEI), decisional conflict (DCS), and depression (EPDS) were calculated for both groups (Table4). Statistically significant differences in fear (*p *<* *0.001) and childbirth confidence (*p* = 0.002) were found between groups (Bonferroni significance levels) 32. Although there was a reduction in decisional conflict and depression symptoms, there was no statistically significant difference (*p* = 0.09; *p* = 0.38, respectively) between groups (Table4).

**Table 4:** Change in Scores for Intervention and Control Groups for Primary and Secondary Outcomes

Variables	Intervention *N *= 101		Control *N *= 97		Change in scores			
Primary and secondary outcomes	*n*	Mean change score [SD]	*n*	Mean change score [SD]	Mean change difference	95% CI for mean change difference	*p*	Effect size
WDEQ	98	19.52 [18.59]	96	9.28 [16.32]	10.24	5.29–15.19	< 0.001*	0.59†
CBSEI	97	61.10 [87.45]	91	19.70 [92.61]	41.40	15.48–67.30	0.002*	0.46†
DCS	101	21.74 [25.69]	95	16.13 [21.24]	5.60	−1.06–12.26	0.09	n/a
EPDS	101	1.26 [4.98]	97	.61 [5.30]	.65	−0.79–2.09	0.38	n/a

* Bonferonni indicates significance at *p* = 0.0125. Change scores were calculated by subtracting time 2 from time 1 (WDEQ, DCS, EPDS) and for CBSEI subtracting time 1 from time 2.

† Moderate effect size 32. W-DEQ = Wijma Delivery Expectancy Scale; CBSEI = Childbirth Self-Efficacy Inventory; DCS = Decisional Conflict Scale; EPDS = Edinburgh Postnatal Depression Scale.

To date, there is insufficient evidence to determine if fear naturally increases or decreases across pregnancy; however, a reduction in childbirth fear scores were found for women in the control group at 36 weeks compared to baseline scores in this study (Control Group: Mean W-DEQ A: 75.7 to 66.5). A 20-point difference in W-DEQ scores delineates high (W-DEQ ≥ 66), severe (W-DEQ ≥ 85), and extreme childbirth fear (W-DEQ ≥ 100) 2,10. Therefore, a change in 20 points would indicate a clinically meaningful change because of decreasing severity of childbirth fear, and was chosen for chi square analysis to determine the percentage of participants who improved versus no improvement. More women in the intervention group showed improved childbirth fear scores (*n* = 48/98, 49%) compared to controls (*n* = 25/96, 26%), (χ^2^(1, *n* = 194) = 9.92, *p* = 0.002, phi = −.3) as shown in Table5. Based on Cohen's criteria 33 a moderate effect was seen.

**Table 5:** Percentage of Participants in Intervention and Control Groups Who Showed Improvement on Primary and Secondary Outcomes

Outcome variables	Intervention *n* = 101 No. (%)	Control *n* = 97 No. (%)	*p*
Fear (WDEQ-A)			
Improved > 20 score	48 (47.5)	25 (25.8)	0.002
No Improvement	50 (59.5)	71 (73.2)	
Missing	3 (3)	1 (1)	
Childbirth confidence (CBSEI)			
Improved	74 (73.3)	55 (56.7)	0.03
No improvement	23 (22.8)	36 (37.1)	
Missing	4 (4)	6 (6.2)	
Decisional Conflict (DCS)			
Improved > 12 score	66 (65.3)	50 (51.5)	0.09
No Improvement	35 (34.7)	45 (46.4)	
Missing	–	2 (2.1)	
Depression (EPDS)			
Improved	56 (55.4)	47 (48.5)	0.40
No Improvement	45 (44.6)	50 (51.5)	
Missing	–	–	

W-DEQ = Wijma Delivery Expectancy Scale; CBSEI = Childbirth Self-Efficacy Inventory; DCS = Decisional Conflict Scale; EPDS = Edinburgh Postnatal Depression Scale.

A 12-point or more improvement in DCS score was adjusted for, being the minimal score difference between making or delaying decision making. No adjusted changes were made to CBSEI or EPDS scores. A higher percentage of women who received the intervention had higher levels of childbirth confidence (CBSEI) (*n* = 74/97, 76.3%) compared to controls (*n* = 55/91, 60.4%), (χ^2^(1, *n* = 188) = 4.8, *p* = 0.03, phi = −3) with moderate effect. Although not significant, reduced levels of decisional conflict were found for women who received the intervention (*n* = 66/101, 65.3%) compared to controls (*n* = 50/95, 52.6%), (χ^2^(1, 96) = 2.8, *p* = 0.09, phi = −.2) with small to moderate effect 33. Similarly, a higher percentage of women in the intervention group showed improvement for depression (*n* = 56/101, 55.4%) compared to controls (*n* = 47/97, 48.5%); however, this improvement was not statistically significant, (χ^2^(1, *n* = 198) = .71, *p* = 0.40, phi = 0.15 (Table5).

## Discussion

This brief psycho-education telephone counseling intervention offered by midwives during pregnancy was effective in reducing women's fears, and improved childbirth confidence. Two previous studies of antenatal interventions by midwives to reduce childbirth fear showed reduction in requests for cesarean. Neither study, however, used a randomized controlled trial (RCT) design to evaluate the intervention, nor did they use a standardized measure of fear 18,34.

The closest comparative studies are two RCTs conducted in Finland. Saisto et al randomized fearful nulliparous and multiparous women deemed at low obstetric risk to receive either six counseling sessions or two sessions provided within conventional care 19. The intervention was delivered by an obstetrician trained in cognitive psychotherapy. A significant reduction in women's birth concerns was found in the intervention group, with decreased requests for cesarean in both study groups. A more recent RCT by the same team offered women with severe fear (WDEQ ≥ 100) six antenatal and one postpartum group sessions with a psychologist (14 hours overall). The intervention reduced fear levels, and improved vaginal birth rates 20 and confidence for women receiving group psycho-education 35. Although 56 percent of women in their control group also received specialized support for fear from other services, women allocated to the intervention group fared better.

The interventions by the Finnish researchers had similar intent to the current study to increase women's knowledge and confidence by identifying women's feelings that affect choices for their pending births. However, Saisto et al did not measure fear using the WDEQ-A, and Rouhe et al did not measure fear in the third trimester 19,20. Furthermore, women in control groups of both studies had access to other treatment options for fear. In the current Australian maternity system, no specific treatment for childbirth fear is available, and this allows for a clear determination that differences were a result of the midwife intervention and not confounded by women accessing a similar service elsewhere.

### Childbirth Confidence

Low self-efficacy is associated with childbirth fear, increased perception of pain in labor, and obstetric interventions 36,37. We found that a brief midwife telephone counseling intervention for women with high childbirth fear could significantly improve confidence in women of any parity during pregnancy, and confirmed that childbirth self-efficacy is modifiable. A recent Australian pilot study also found significant improvement in women's (*n* = 18) childbirth self-efficacy and fear levels following a mindfulness antenatal education program with first-time mothers 38. That program included 20 contact hours with an experienced yoga and childbirth educator and required participants to complete a precourse homework package. An efficacy enhancing antenatal education program conducted in China also reported improved childbirth confidence and lower perception of pain and anxiety during labor 39. Two studies in Taiwan improved childbirth confidence through the use of birth balls in labor 40 and a prenatal yoga program 41. These three studies, however, did not include parous women or women with childbirth fear. Our study is the first RCT to test childbirth self-efficacy within an Australian population of fearful women.

Improving women's belief in their ability to cope with normal physiological and emotional challenges of labor is fundamental to birth preparation. The task is even more challenging in women who are fearful of birth and at higher risk of requesting a cesarean to avoid the experience of labor.

Women in our cohort who had lower incomes and education levels were less likely to continue in the study. Further investigation is required to determine the best methods for engagement and providing support. Women's lack of confidence may have impacted their ability to continue participation, or challenging women's health beliefs may have contributed to attrition and would be best assessed and monitored through a trusted continuity of caregiver relationship.

### Decisional Conflict

Women receiving the BELIEF intervention reported lower levels of decisional conflict than controls at 36 weeks of pregnancy but the change was not significant. This finding may be an important one. High rates of decisional conflict are associated with regret and ambivalence 42 and may have consequences for women's birth choices and satisfaction. Decisional conflict is commonly measured in studies investigating women's decision making in a subsequent pregnancy after a previous cesarean and has not been applied to childbirth fear 43,44. Our participants with childbirth fear had higher baseline decisional conflict scores compared to women in other studies who were possibly less distressed and making choices about birth mode alone. The reduction in decisional conflict in both the intervention and control groups may indicate not only the benefit of the decision aid for women with childbirth fear but also that psycho-education with a midwife enhances the effectiveness of the decision aid.

### Depression Symptoms

Poor emotional health is associated with increased childbirth fear 13,15,45. In our study, 21 percent of women reported EPDS scores of greater than 12, which is higher than the 8.1 percent identified in fearful Norwegian women (WDEQ ≥ 85) 46 but lower than the 30 percent rate of probable depression in fearful Swedish women 47. Our telephone counseling intervention reduced women's EPDS scores compared to baseline, but not significantly. Positive effects may be attributed to listening and facilitating women's concerns, where their worries were addressed and alleviated. The reduction in depressive symptoms following the intervention in the current study aligns with previous advice to explore women's unhappiness in the preparation for birth 48. This finding also gives support for midwifery continuity of care models where psychosocial issues are more readily addressed than within biomedical models of care 11,49,50.

### Limitations

Recruitment occurred within the public health system; however, one-third of all women in Australia receive private health care. At baseline, demographics were similar to the national birthing population, but of those randomized, women who were less educated and poorer were less likely to continue in the study. Women requiring an interpreter were also not included; therefore, caution is needed in drawing generalizations. Although there may be differences in childbirth fear by parity, the sample size was not sufficient to support subgroup analysis.

However, the robustness of findings is strengthened because of the RCT design with participants stratified by site and parity. The study was the first to include a measure of decisional conflict and therefore no comparison to other childbirth fear studies could be made. Investigating decisional conflict in this group of vulnerable women can shed light on how and why they make health decisions, and increase awareness about how informed consent for procedures might be determined. Although conducting the intervention over the telephone offered a great deal of flexibility and accessibility for participants, there were also the disadvantages. A small proportion of women were multitasking (for example doing dishes, washing clothes) when receiving the psycho-education. Outcomes may have been further improved had all women been able to find a quiet space for these conversations.

Few women scored extremely high on the W-DEQ (≥ 85, *n* = 42), indicating severe fear with probable clinical symptoms 51. We cannot know whether the BELIEF intervention would be enough in these cases. On the other hand, perhaps reducing moderate fear in one pregnancy may prevent a development of a more serious childbirth anxiety or phobia later, in the same or in the next pregnancy. Furthermore, a proportion of women did not proceed with the intervention. Engagement of fearful women may have been enhanced by using other modes such as the Internet, which would allow 24-hour access to educational materials and chat options for questions and support.

## Conclusions

This was the first reported RCT of an antenatal intervention for childbirth fear by midwives. The BELIEF psycho-education intervention facilitated a communicative and caring partnership with women to reduce or heal childbirth fear from mid to late pregnancy. Our results indicate fear can be modified. Assisting women to reframe their perceptions about their ability to birth is a critical strategy in preserving the normality of birth. Provision of educational and emotional support has the potential to reduce interventions such as cesarean delivery and the emotional or psychological consequences of what could be otherwise experienced as a disappointing or traumatic birth 52. Assisting women to achieve a normal birth will improve women's quality of reproductive life, reduce health care costs, and improve postpartum maternal and child health outcomes 21,28.

Asking women explicitly about their fears and concerns significantly lowered fear and improved childbirth confidence compared to women receiving standard care. Consideration needs to be given to routine screening around childbirth fear, and application of existing best evidence for promoting normal birth. There was a tendency toward improved women's decision making with the provision of a decision aid and access to individualized midwifery telephone support; however, the difference was not statistically significant. This tendency has important implications for how informed consent is secured in clinical practice and within the context of a maternity culture of high obstetric interventions where women's ability for ready decision making is shown to be vulnerable. Furthermore, the intervention provided a midwife confidante with whom women could share and discuss their concerns, and have their concerns heard. This level of information and support may be particularly important where women are not offered continuity of care or where social support is lacking.

The BELIEF telephone intervention is brief (particularly in comparison to other reported interventions), reproducible (given it has been adapted from use with postnatal women) 28, effective, and could be easily introduced into routine midwifery practice with women reporting high fear. BELIEF involves listening and responding to women's feelings (a factor previously identified as important) and provides consistent and accurate information. The current intervention is adaptable for individual sessions or group work, and could be delivered in person or using other media. Given the geographical remoteness of some health services in Australia and other countries, flexibility in delivery is important. Where trusting relationships are built, women are more likely to stay engaged in care.
